# Gut-Centric Multi-System Regulation by *Bacillus subtilis* and *Bacillus natto*: A Review of Their Probiotic Functions in Nutrition, Immunity, and Metabolism

**DOI:** 10.3390/nu18050802

**Published:** 2026-02-28

**Authors:** Mei Hua, Jing Wang, Yueqiao Li, Yuguang He, Zhengyang Luo, Da Li, Mubai Sun, Xinyu Miao, Honghong Niu, Tong Pan, Jinghui Wang, Chengshan Wan

**Affiliations:** 1 Institute of Agro-Food Technology, Jilin Academy of Agricultural Science (Northeast Agricultural Research Center of China), Changchun 130033, China; huamei@jaas.com.cn (M.H.);; 2 College of Agriculture, Yanbian University, Yanji 133002, China; 3 Institute of Resources and Environment, Jilin Academy of Agricultural Science (Northeast Agricultural Research Center of China), Changchun 130033, China

**Keywords:** *Bacillus subtilis*, *Bacillus natto*, spore-type probiotics, nutrient absorption, intestinal microecology, immune system

## Abstract

Background: Compared with lactic acid-producing probiotics, spore-producing probiotics such as *Bacillus subtilis* (BS) and *Bacillus natto* (BN) exhibited superior metabolic capacity and stress resistance and are more suitable for industrial applications. However, limited understanding of their nutritional and intestinal health mechanisms has constrained their food potential. Objectives: This review systematically expounded on the ‘triple mechanism’ of BS and BN and their effects on intestinal nutrition, immunity and metabolism benefit for the first time. Methods: We searched PubMed, Scopus, Web of Science, and Google Scholar for studies on livestock, model organisms, and human research from 2000 to 2025. After evaluating relevance and eligibility, 115 articles were included. Results: Firstly, by secreting various digestive enzymes, BS and BN directly enhanced the small intestine digestive and absorptive efficiency and promoted animal growth. In particular, BN significantly increases calcium absorption in postmenopausal women. Secondly, as the antigen carrier that induced intestinal mucosal immunity, BS and BN enhanced the host’s defense ability by strengthening the expression of tight junction proteins, mucins, and inflammatory factors and bidirectionally regulated constipation and acute diarrhea in the human body. Thirdly, they reshaped the structure of the intestinal microbiota and their metabolic profile in the form of the gut–liver/gut–adipose axis, including enriching beneficial bacteria, activating lipid metabolism pathways such as PI3K/AKT and AMPK/SREBP, and regulating liver targets such as PPAR and CD36, thereby reducing insulin resistance and liver injury and maintaining overall metabolic homeostasis. Conclusions: *Bacillus subtilis* and *Bacillus natto* mediated their probiotic benefits through a gut-centric, multi-system regulatory strategy, involving nutrient utilization, immune homeostasis, and microbial–host metabolic interactions. This integrated mechanism provided a robust foundation for their targeted application in functional formulations and fermented food science.

## 1. Introduction

Gut microbiota, comprising bacteria, viruses, fungi, and other microorganisms residing within the gastrointestinal tract (GIT), are increasingly recognized as a virtual organ essential to human health. Substantial research demonstrates their critical role in the pathogenesis and progression of numerous chronic diseases, including inflammatory bowel disease, obesity, type 2 diabetes, cardiovascular disease, and cancer [1]. ‘Probiotics’ are defined as live microorganisms that, when administered in adequate quantities, confer a health benefit on the host [2]. Internationally recognized genera currently include *Lactobacillus*, *Bifidobacterium*, yeasts (e.g., *Saccharomyces boulardii*), and *Bacillus* [3]. Unlike common probiotics such as *Bifidobacterium*, *Lactobacillus*, and many yeasts, which are often sensitive to environmental stressors like light and temperature, *Bacillus* species possess a unique protective endospore structure. This spore form confers significant resistance to damage encountered during conventional food processing and storage, establishing *Bacillus* probiotics as key representatives of novel stress-tolerant probiotics [4].

Consequently, several *Bacillus* species are now utilized across pharmaceutical, food, and animal husbandry industries. For instance, *Bacillus clausii* has demonstrated efficacy in clinical trials by assisting immunoglobulin A (IgA) synthesis and modulating the human immune system [5]. *Bacillus coagulans* received Generally Recognized as Safe (GRAS) status from the US Food and Drug Administration (FDA) for food and pharmaceutical applications in 1992. It was subsequently approved by China’s National Medical Products Administration (NMPA) for treating intestinal disorders in 2005 and was included in China’s ‘List of Bacteria Permitted for Use in Food’ in 2016 [6]. Clinical evidence indicates that supplementation with *B. coagulans* for 30 days enhanced immune responses and reduced susceptibility to viral infections, including adenovirus and influenza A virus [7].

*Bacillus subtilis* (BS) is recognized as the ‘perfect probiotic’ for human and animal intestines owing to its robust colonization capacity, efficient nutrient utilization, multi-enzyme secretory activity, and abundant metabolite production [8]. *Bacillus natto* (BN), a subspecies of *Bacillus subtilis*, is among the 40 probiotic strains certified by the US FDA [9]. BN synthesizes unique bioactive compounds including nattokinase, vitamin K_2_, and pyrroloquinoline quinone (PQQ), which demonstrates beneficial effects in enhancing nutrient digestion/absorption, improving intestinal microecology, and modulating immune function [10]. Consequently, BN is extensively utilized in functional foods targeting thrombosis dissolution, blood lipid reduction, cardiovascular/cerebrovascular health improvement, and intestinal homeostasis [11].

**Figure 1:**
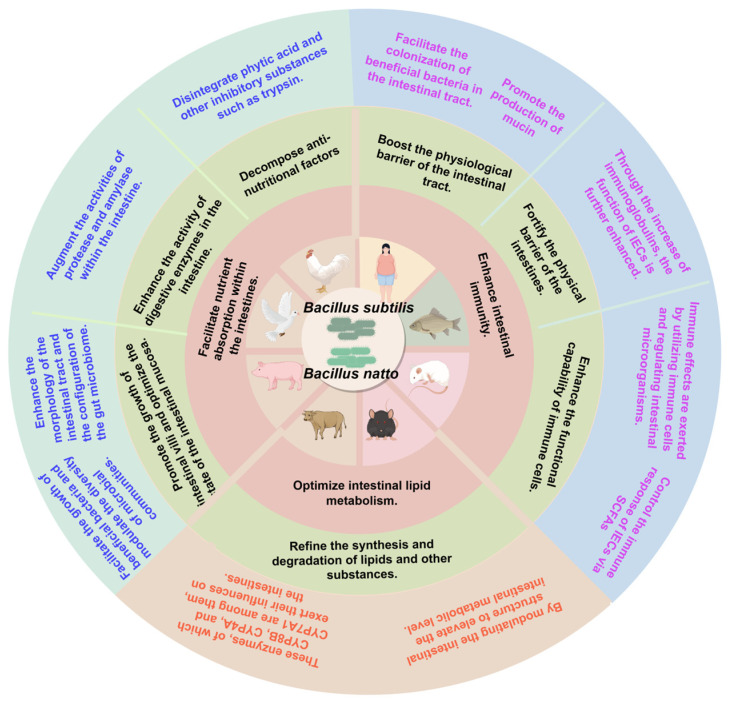
The beneficial effects of *Bacillus subtilis* and *Bacillus natto* in intestinal nutrient absorption, intestinal microecological balance, and intestinal immunity.

Presently in China, BS is primarily applied in animal husbandry and environmental sectors such as in microecological preparations, antibacterial agents, and water quality amendments. However, as calls for including BS and BN in edible probiotic registries intensify, exploration and functional validation of their probiotic characteristics have garnered significant scientific interest. This review systematically synthesizes research on BS and BN concerning probiotic functions in intestinal nutrient absorption, microecological balance, immune regulation, and metabolic modulation across livestock, poultry, model animals, and humans. The synthesis aims to establish a theoretical foundation for advancing Bacillus-based probiotics and fermented foods (Figure 1).

## 2. The Effects of *Bacillus subtilis* and *Bacillus natto* on Intestinal Nutrient Absorption

### 2.1. BS and BN Facilitate Intestinal Nutrient Absorption in Livestock and Poultry Animals

In China, BS has been extensively employed as a feed additive to improve nutrient utilization and growth performance in livestock. Mechanistically, BS mitigated the effects of anti-nutritional factors (e.g., phytic acid and trypsin inhibitors) by secreting digestive enzymes, enhancing intestinal villus architecture, and modulating the gut microbiota [12]. Studies have found that BN diet increased the intestinal trypsin, amylase, lipase and total protease activities of Ross 308 chicks, reduced serum ammonia, uric acid, total cholesterol (TC) and triglyceride levels, and promoted the growth. BN promoted the nutrient absorption of intestinal epithelial cells (IECs) by upregulating the expression of jejunal sodium/glucose cotransporter-1 (SGLT-1), glucose transporter-2 (GLUT-2) and peptide transporter-1 (PEPT-1) [13,14]. After eating BS feed for 42 days, broiler chickens showed higher growth performance, which was related to the high expression of mucin-2 (MUC2) gene in goblet cells and the morphological changes in small intestine [15]. Another study found that after 42 days of BN feed feeding, the activities of protease and amylase in the pancreas and in the duodenum and jejunum of broiler chickens increased, and the villus height and the ratio of villus height to crypt depth (VH/CD) improved. The intestinal apoptosis index (AI) is significantly reduced, and the intestinal nutrient absorption efficiency of broiler chickens is improved [16]. After feeding 4 different BS feed to red sea bream for 60 days, their final body weight (FBW), weight gain (WG), growth rate (GR), feed conversion efficiency (FCE) and protein efficiency ratio (PER) were significantly improved. This may be due to the fact that BS feed increased the activity of digestive enzymes in the fish intestine and the abundance of beneficial bacteria such as *Lactobacilli* [17,18]. Comparable outcomes were observed in grouper and white-leg shrimp, wherein BS improved intestinal structure, upregulated prophenoloxidase and serine protease gene expression, and boosted digestive enzyme activities [19,20,21,22,23].

BS and BN also regulate the structure of the intestinal flora via competitive inhibition, thereby enhancing the growth performance of livestock and poultry. In White King pigeons, BS altered the fecal Firmicutes/Actinobacteria/Bacteroidetes ratio, increasing the abundance of *Enterococcus faecalis*, *Enterococcus*, and *Bifidobacterium* [24,25]. Feeding on HC6 (BS) diet for 50 days increased the relative abundance of *Bacteroidales_unclassified* and *Olsenella* in the intestinal tract of white-feather broilers and improved their jejunal feed efficiency. This change is significantly related to high feed conversion rate and the expression of jejunal mucosal barrier genes ZO-1, claudin-1 and occludin [26,27]. After feeding Bamei piglets with BS QB8a-fermented feed for 28 days, their diarrhea rate and growth performance were improved. This effect was related to increasing serum alkaline phosphatase (AKP) and transpeptidase (TP) activities and the abundance of *Prevotellaceae* and *Rikenellaceae* in intestine [28,29]. BN-710 feed inhibited the blood ammonia concentration of male white leghorn chickens (*Gallus gallus domesticus*) by improving the intestinal flora structure, promoting cell mitosis and intestinal villus growth, thereby activating intestinal function and improving nutrient digestion and absorption [13,30]. In Muscovy ducks, BN improved duodenal morphology, suppressed *Escherichia coli* and *Salmonella*, increased *Lactobacillus*, *Bifidobacterium*, and *Faecalibacterium* loads, and effects were mediated by catalase and protease secretion that enhanced protein absorption [31,32]. Adding BS to the diet improved the growth performance of finishing pigs, which was positively correlated with the increase in acetate and butyrate content in their feces [33]. Other studies reached similar conclusions. Adding butyrate to feed increased the ileal villus height and colonic short-chain fatty acids (SCFAs) levels of pigs and maintained the intestinal barrier function by increasing the mRNA expression levels of *protein kinase C*, *claudin-1*, *mucin-1*, and *β1 integrin* in the ileum, promoting piglet growth and improving pork quality [31,34]. Similar results were obtained in a study on nursery pigs [35,36]. BS increased the intestinal glucose absorption by upregulating the mRNA expression levels of GLUT-2, thereby increasing fecal SCFA levels, improving intestinal microbial structure, and increasing pig growth performance [36]. The FCR of neonatal Holstein calves was significantly improved after feeding on BS B27 diet for 83 days, which was attributed to the fact that probiotics BS promoted the proliferation of rumen flora and increased the utilization of nutrients [37]. Similar results were observed in sheep studies. After feeding on BS C-3102 diet for 80 days, the ruminal protein synthesis levels, GR, FE and Volatile Fatty Acid (VFA) of Duhan hybrid lambs increased. This may be related to the increased abundance of *Bacteroidetes* and the elevated butyrate concentration in rumen, which promoted polysaccharide decomposition and cellulose digestion [38,39].

In vitro, BN fermentation significantly improved protein digestibility and eliminated anti-nutritional factors. Compared with cooked or autoclaved soybeans, BN-fermented soybeans exhibited a 45% increase in protein digestibility, elevating absorption rates from 50% to over 90% [40]. In vitro digestion experiments also showed that sea cucumber polysaccharides fermented by BN increased the abundance of polysaccharide, degrading bacteria *Acinetobacter* and improving the carbohydrate digestibility of the intestine [41].

Collectively, these findings demonstrated that BS and BN potentiated intestinal nutrient absorption in livestock, poultry, and aquatic species through a multi-target mechanism involving digestive enzyme secretion, nutrient transporter upregulation, intestinal barrier reinforcement, and gut microbiota modulation. By concurrently improving growth performance, feed efficiency, and metabolic profiles, BS and BN represent efficacious and sustainable probiotic feed additives with substantial potential for broad agricultural application.

### 2.2. BS and BN Enhance Intestinal Nutrient Absorption in Model Animals

Due to their powerful enzyme system and intestinal survival rate, the application of BS and BN in disease model animals has attracted increasing attention. The scholars used BALB/c mice as model animals to study the effect of poly(γ-glutamic acid) (γ-PGA) produced by BS on intestinal calcium solubility. The results showed that as the γ-PGA content increased, the intestinal calcium utilization rate of mice significantly increased, and the insoluble complex formed by calcium and phosphate was inhibited [42]. BS also ameliorated intestinal dysfunction by modulating bile acid metabolism and activating the Takeda-G-protein-receptor-5 (TGR5) pathway. BS promoted the release of serotonin (5-HT) from enterochromaffin cells (ECs), thereby mitigating colonic pathological damage, enhancing intestinal peristalsis, and ultimately improving both intestinal nutrient absorption and host health [10]. Similarly, gavaging BS (R0179) to C57BL/6 mice promoted their growth. The tryptophan produced by BS affected the 5-HT signaling pathway in the intestinal epithelial layer and increased intestinal peristalsis [43]. In addition, BS also promoted intestinal digestion in animals by maintaining the balance of intestinal flora. BS-18 increased the average daily WG of KM mice, reduced their FCE, and improved the morphology of intestinal mucosa. The abundance of beneficial bacteria such as *Lactobacillus* in the duodenum and jejunum increased significantly, which is related to BS-18’s good digestive performance [44]. Similarly, after 30 days gavage of BS (KT 260179) to KM mice, the WG and the abundance of beneficial bacteria *Lactobacillus* and *Bifidobacterium* in cecum significantly increased, and the abundance of harmful bacteria *E. coli* and *Staphylococcus aureus* significantly decreased. Intestinal mucosal morphology and the ratio of villus height to crypt depth (V/C) were also improved, and the intestinal flora structural changes were closely related to the nutrient absorption efficiency [45].

In addition, BN not only promoted the intestinal colonization of beneficial bacteria by competing with harmful bacteria for nutrients but also maintained the digestive activity of probiotic bacteria in the intestine [46]. After feeding KM mice with BN-fermented peanut meal (PM) for 4 weeks, the daily body mass index in the high-dose group significantly increased. This was consistent with the changes in the intestinal flora structure and amino acid utilization caused by BN-fermented feed [47]. Moreover, feeding BN fermentation broth rich in γ-PGA significantly increased the soluble calcium content in the intestinal contents of rats [48], which supported the previous conclusion that BS secretes γ-PGA to promote calcium absorption [42]. This convergence suggested that BS and BN may regulate intestinal nutrient uptake through overlapping mechanisms centered on γ-PGA production and microbiota remodeling.

Collectively, these studies in model animals established that BS and BN enhanced intestinal nutrient absorption through integrated mechanisms involving γ-PGA-mediated calcium solubilization, bile acid–TGR5–5-HT signaling, and targeted modulation of the gut microbiota. By improving mucosal morphology, suppressing pathobionts, and enriching beneficial commensals, both probiotics effectively optimized host nutrient utilization and intestinal health. These findings provided a mechanistic rationale for the translational application of BS and BN in functional foods and therapeutic interventions for malabsorption syndromes.

### 2.3. BS and BN Promote Intestinal Nutrient Absorption in Humans

Although BS has been widely proven to improve the growth performance of livestock and poultry animals, there are few studies on its impact on humans. The high-throughput SIFR^®^ (Systemic Intestinal Fermentation Research) technology was used in a study to evaluate the impact of functional foods added with BS HU58™ on human intestinal microbiota [49,50]. The results showed that GoodBiome™ foods (BBM/LCM/OSM) not only promoted small intestinal digestion but also increased the SCFAs content greatly, which were related to the increased abundance of *Bifidobacteriaceae* and *Bacteroidetesaceae*. In addition, although natto has been consumed in Asia for more than 1000 years, current research on its human health effects is still limited, and only a few ingredients such as γ-PGA have been verified. An investigation on natto γ-PGA consumption in postmenopausal women showed that a single dose of γ-PGA significantly increased the calcium absorption rate in the small intestine [51]. This effect may be related to the activation of the MCM3-Keap1-Nrf2 signaling pathway in osteoblasts and the upregulated expression of fibrillin-1 (Fbn1) gene. It is also related to the inhibition of oxidative stress and osteoclast bone resorption and the promotion of osteoblast bone formation [50,52]. The above study on the role and mechanism of BS and BN in promoting nutrient absorption in animals and humans are summarized in Table 1. These findings underscored the translational potential of BS and BN as functional food constituents for improving human nutrition and mitigating age-related bone loss. Further randomized controlled trials are warranted to substantiate these benefits across broader populations.

## 3. Effects of *Bacillus subtilis* and *Bacillus natto* on the Intestinal Immune Function

### 3.1. BS and BN Fortified the Intestinal Immune Function of Livestock and Poultry Animals

Intestinal immunity includes innate immunity, adaptive immunity and microbe–host interaction. The entire intestinal mucosal system is composed of mucus layer (physiological barrier), epithelial layer (physical barrier) and numerous immune cells. Intestinal immune function is mainly realized based on the above structure [53].

#### 3.1.1. Fortification of the Intestinal Physiological Barrier

As the first line of defense for the intestinal physiological barrier, the mucus layer prevents bacteria from directly invading IECs. Probiotics can adhere to IEC receptors through adhesins, occupy and colonize, inhibit intestinal colonization of pathogenic microorganisms, and exert the biological barrier function [54]. After feeding BS to healthy laying hens for 50 days, the mRNA expression level of the tight protein occludin in the duodenum was increased [55]. In Nile tilapia, eight weeks of BS feeding increased the number of intestinal epithelial lymphocytes (IELs), bolstered mucosal immunity, and preserved intestinal homeostasis [56]. In addition, surface proteins of BN mediated intestinal mucus adhesion in *Paralichthys olivaceus* during *Vibrio anguillarum* infection, facilitating probiotic colonization and restoring the intestinal microecological environment [57].

#### 3.1.2. Strengthen the Intestinal Physical Barrier

IECs are considered an important physical barrier of the intestinal mucosa, capable of directly sensing information related to intestinal pathogens and coordinating necessary immune responses. Goblet cells in IECs can assist beneficial bacteria in completing intestinal colonization and prevent microorganisms from entering the IECs [58]. Supplementing BS in laying hens’ diets improved the intestinal barrier integrity, significantly increased the number of ileal goblet cells and the levels of immunoglobulin G (IgG), and enhanced the intestinal secretory immunoglobulin A (sIgA) levels in a dose-dependent manner [59]. Supplementing *B. subtilis* DSM 32540 to piglets not only expanded the number of goblet cells, sulfomucin percentage in duodenal villi and sialomucin percentage in jejunal villi but also downregulated the mRNA expression levels of PTGS2 and IL1B in ileal mucosa, maintaining the intestinal barrier integrity [35]. Similar effects were observed in Cherry Valley ducks. Compared with the normal group, the number of goblet cells in the jejunal mucosa of the BS (PB6)-supplemented group was increased, and the mRNA expression levels of barrier protein (ZO-1) and ileal mucin-2 were significantly upregulated [60].

#### 3.1.3. Enhancement of Immune Cell Function

Beyond the physiological barrier of the mucus layer and the physical barrier of IECs, 80% of the intestinal immune system exists in the gastrointestinal tract in the form of gut-associated lymphatic tissue (GALT) and exerts effects through immune cells [54]. Among them, dendritic cells (DCs) are the most effective antigen-presenting cells (APCs). They are mainly responsible for recognizing and presenting antigens to lymphocytes and inducing antigen-specific immune responses by regulating tolerance and immunity to microbial antigens [61]. A large number of livestock and poultry animal studies have proven that BS can directly increase the phagocytic function of macrophages [62]. Other studies have confirmed that BN can increase the number of macrophages and DCs in the intestinal lamina propria, thereby enhancing host intestinal immune function [63]. In vitro, BN-derived spores promoted the production of Th1-type cytokines by downregulating the mRNA expression of Toll-like receptor 4 (TLR4) and upregulating the mRNA expression of Toll-like receptor 2 (TLR2), myeloid differentiation factor 88 (MyD88) and nuclear factor kappa B (NF-κB), and induced macrophage M1 polarization through the TLR2 [64]. Comparative analysis of 23 BN isolates from natto products revealed that chaperone proteins in BN fermentation supernatants, following 20 h incubation with THP-1 DCs, significantly modulated IL-10 and IL-12 expression through TLR2-dependent pathways [62]. Additionally, BS enhanced chicken T cell activation and proliferation in vitro [65]. In Nile tilapia, dietary-inactivated BN improved growth performance and intestinal microbial composition while inducing immunomodulatory metabolites—including palmitic acid and L-tyrosine—that actively participated in intestinal immune responses [66].

#### 3.1.4. Regulation of the Interactions Between the Host and Gut Microbiota

Host–microbe interactions play a key regulatory role in intestinal mucosal immunity. Its primary mechanism is believed to involve SCFAs, important metabolites of intestinal flora. SCFAs can directly control the immune response of IECs by regulating downstream signaling pathways through G protein-coupled receptors (GPCRs) such as GPR41, GPR43, and GPR109A and so on. BN has been confirmed to improve intestinal immune function by regulating host–microbe interactions [67]. After feeding BN feed to chickens for 80 days, the cecal acetic acid concentration increased, acetate entered the peripheral circulation and was metabolized by peripheral tissues, and the chicken immune function was enhanced [68]. Butyrate is the main energy source for intestinal cells and an important intestinal immune modulator. Research has demonstrated that supplementing broiler diets with sodium butyrate (SB) for 42 days results in a significant increase in the abundance of butyrate-producing bacteria, including *Ruminococcus*, *Lachnoclostridium*, *Anaerostipes*, and *Butyricicoccus* [69].

Collectively, BS and BN fortified intestinal immunity in livestock and poultry through a multilayered defense strategy encompassing: (i) reinforcement of the mucus and epithelial barriers via tight junction proteins and goblet cell expansion; (ii) activation and polarization of macrophages, DCs, and T cells through TLR2/NF-κB signaling; and (iii) metabolic modulation of host–microbe interactions via SCFAs and other bioactive metabolites. These integrated mechanisms effectively protected the intestinal mucosa from pathogenic invasion and sustained immune homeostasis, positioning BS and BN as potent, non-antibiotic immunomodulatory feed additives for sustainable animal production.

### 3.2. The Effects of BS and BN on Intestinal Immunity in Model Animals

In model animals, the intestinal immune effects of BS were mainly accomplished by stimulating the expression of immune cytokines, enhancing the intestinal barrier, and modulating inflammatory factors. Moreover, the local immune response in the intestine also alters the immune level of distal intestinal organs [70]. In *Citrobacter rodentium*-infected mice, BS effectively safeguarded the intestinal mucosa, regulated the TLR2/NF-κB and TLR2/MAPK signal pathways, suppressed the host inflammatory response, and preserved intestinal barrier integrity and immune homeostasis [71]. In addition, as a safe and efficient non-pathogenic bacterial carrier, BS delivered antigens to the intestinal mucosa in the form of vegetative cells or spores. Upon ingestion, BS spores germinated within the gastrointestinal tract and expressed target antigens either in the intestinal lumen or within phagocytic antigen-presenting cells (APCs), thereby eliciting specific antibody responses in both serum and mucosa [72].

The immunomodulatory effect of BN has also been confirmed in model animals. BN B4 spores enhanced the immune function of mice by increasing the activities of acid phosphatase (ACP), lactate dehydrogenase (LDH), and inducible nitric oxide synthase (iNOS) in cells, and improving the expression level of macrophage inflammatory protein-2 (MIP-2) [73]. Both *Bacillus subtilis* BS02 and *Bacillus natto* BS04 enhanced the phagocytosis function of the mononuclear phagocyte system (MPS) and the cytotoxicity of natural killer cells (NK). BN BS04 also increased the percentage of CD3^+^ cells and CD3^+^ CD4^+^ cells in splenocytes, enhanced T cell responses, reduced the ability of pathogens to escape the immune system, and directly induced Th1 activation and systemic immunity reaction in BALB/C mice [74,75].

**Figure 2:**
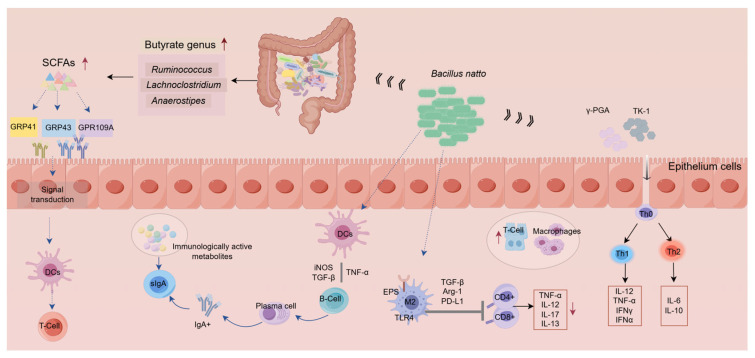
Potential mechanisms of *Bacillus subtilis* and *Bacillus natto* and their effects on intestinal immunity. The red arrow indicates an increase or decrease in abundance or expression level, while other arrows represent indication or connection.

BN also participates in intestinal immunity by regulating the structure of intestinal flora. BN increased the abundance of *Lactobacillus*, *Faecalibacterium*, and *Clostridium butyricum*, for which the *Clostridium butyricum* can reduce the protein expression level of NF-κB, reduce the inflammatory response induced by LPS, and enhance the intestinal barrier function [76]. Furthermore, γ-PGA, mainly generated by BS, induced the expression of IL-12, CD80 and CD86 in DCs and macrophages in a TLR4-dependent manner, promoted DCs which drive the polarization of T_h_1 cells, and also affected T_h_17 cell development in a APCs-independent manner [77]. Similarly, surfactants produced by *Bacillus natto* TK-1 not only significantly reduced the levels of IFN-γ, IL-6, iNOS, and nitric oxide (NO) but also downregulated the expression of TLR4 protein in LPS-induced macrophages, indicating that BN may exert its immune effects by affecting inflammatory factors and TLR4-related signaling pathways. Further studies have also confirmed that BN-secreted surfactants blocked LPS-stimulated macrophages from producing TNF-α, IL-1β and NO by inhibiting NF-κB activation and then exhibited anti-inflammatory activity [78]. The potential mechanisms of BS and BN and their effects on intestinal immunity are summarized in Figure 2.

In model animals, BS and BN orchestrated multifaceted intestinal immune responses through: (i) direct activation of TLR2/NF-κB and TLR2/MAPK cascades; (ii) induction of M2 macrophage polarization and regulatory T-cell responses; (iii) enhancement of NK and cytotoxic T-cell activity; (iv) antigen delivery and mucosal antibody induction; and (v) microbiota-mediated anti-inflammatory signaling. These interconnected mechanisms collectively reinforced intestinal barrier integrity, suppressed pathogenic inflammation, and established systemic immune competence.

### 3.3. The Function of BS and BN in Human Intestinal Immunity

Existing research on the effects of *Bacillus* sp. on the human immune system is still lacking. BS is currently mainly used as an antidiarrheal agent in the clinical treatment of human anti-infection [79]. Studies have shown that daily consumption of BS not only enhanced the bowel regularity of healthy men and women (23 ± 3.9 years aged) but also had a significant effect on patients with acute diarrhea, and its effect was better than that of *Lactobacillus* [80]. In vitro and model animal studies also suggested that BS may improve the human intestinal immune system by producing substances such as antimicrobial peptides, exopolysaccharides, and quorum sensing factors as well as by modifying the intestinal flora structure beneficially [81,82]. Commercial probiotic formulations developed by Deerland Probiotics and Enzymes (Chicago, IL, USA) included Bss-19, DE111^®^, SG188 and other strains of *Bacillus* sp. These strains have been shown to be able to alter the host intestinal flora and stimulate innate immunity by secreting different antibacterial compounds [79]. In a 4-week survey of healthy people, it was found that the anti-inflammatory immune cell population of the *Bacillus subtilis* DE111 group significantly increased in response to LPS stimulation of peripheral blood mononuclear cells (PBMCs) in vitro [83]. In preschool children, once-daily DE111^®^ ingestion reduced the duration of vomiting, hard/loose stools, and overall gastrointestinal discomfort and provided gastrointestinal health benefits [84]. After offering DE111^®^ to male college athletes for 12 weeks during the offseason, their circulating levels of TNF-α decreased, suggesting that they may benefit from skeletal muscle recovery, thereby reducing body damage caused by acute training or competition [85]. In a study of 100 elderly patients aged 60–74 years, it was shown that intermittent use (10 days orally, 18 days off, 28-day cycle, repeated 4 times) of the *Bacillus subtilis* CU1 strain could increase salivary and fecal sIgA levels and reduced the frequency of respiratory tract infections, suggesting that BS CU1 may be a safe and effective strain that can produce immune response stimulation [86].

As the largest mucosal surface layer of the human body, the intestinal epithelium can establish physical and biochemical barriers between the external environment and the host–immune system and maintain intestinal health by regulating mucosal immunity to produce beneficial metabolites [87]. The mechanism of BN’s immune action on the human intestine includes the improvement and strengthening of intestinal mucosal function and the body immune response. Studies have found that BN spores are not only non-cytotoxic but also retain the ability to adhere to IECs by interfering with the LPS/IL-8 signaling pathway and show anti-inflammatory and immune effects [88]. Further studies on Caco-2 colon cancer cells confirmed that active substances in BS and BN cells can bind to the surface-specific receptors of Caco-2 cells, activate downstream IκB and NF-κB transcription factor complexes to increase the levels of IL-6, IL-8 and other cytokines, and enhance human immunity [89].

Despite limited human data, convergent clinical and ex vivo evidence indicated that BS and BN strains enhanced intestinal immunity through four key mechanisms: (i) reinforcement of barrier integrity and IEC-driven cytokine signaling; (ii) induction of mucosal sIgA; (iii) suppression of pro-inflammatory mediators (e.g., TNF-α, IL-8); and (iv) favorable gut microbiota modulation.

## 4. Effects of *Bacillus subtilis* and *Bacillus natto* on Intestinal Metabolism

### 4.1. Intestinal Metabolism Improvements of BS and BN in Livestock and Poultry

The lipid metabolism was affected after broiler chickens were fed with BS dietary supplementation. Following 42 days of BS feeding, marked reductions in abdominal fat coefficient, liver coefficient, and hepatic and serum cholesterol levels were observed, and which may be due to the role of acetyl-CoA carboxylase (ACC, EC 6.4.1.2)—the rate-limiting enzyme of fatty acid synthesis—thereby influencing hepatic lipid metabolism in female broilers [90]. Rapeseed meal (RSM) fermented by *Bacillus subtilis* 87Y also had a significant impact on the intestinal flora and blood biochemical parameters of piglets. The level of fecal *Lactobacillus* in 87Y group was significantly increased, and the level of *E. coli* and *Clostridium perfringens* were reduced. At the same time, the levels of plasma total protein (TP) and albumin (ALB) increased, and triacylglycerol (TG) and low-density lipoprotein (LDL) levels were reduced; these effects were related to 87Y’s promotion for the proliferation of beneficial intestinal bacteria such as *Bifidobacterium* and *Lactobacillus acidophilus* [91].

In the regulation of lipid metabolism, BN mainly affects the biosynthesis and degradation of cholesterol, fatty acids and triglycerides in livestock and poultry animals. After BN fermentation, the dietary fiber content, cholesterol, bile salts, nitrite ion binding and glucose adsorption capacity of millet bran were significantly improved [92]. These changes may be the basis of BN diet for improving lipid metabolism level in livestock and poultry animals. Blood triglyceride levels were reduced and blood calcium levels increased significantly in hens fed with 0.3% TLRI 211-1 [61]. In addition, the BN diet significantly reduced the contents of fecal skatole and ammonia nitrogen (NH3-N), the abundance of *E. coli* and *Clostridium*, the contents of serum TC and high-density lipoprotein, and the enzyme activity levels of liver CYP2A6 and CYP2E1 in TOPIGS pigs. The mechanisms include the following: (a) *E. coli* and *Clostridium* are important bacterial groups involved in tryptophan conversion to skatole. Their inhibition reduced skatole production. (b) BN-enhanced CYP2A6 and CYP2E1 are important proteases that degrade skatole. Their increased enzyme activity promoted skatole degradation. (c) Substances such as nattokinase secreted by BN may promote the level of cholesterol metabolism involved in cytochrome (P450) family members CYP8B, CYP4A and CYP7A1 [93]. In addition, the study also found that synergistic interaction with *B. coagulans* enhanced the ability of BN to reduce skatole, increase antioxidant activity and improve pork meat quality [93], suggesting a possible gain in the effect between different species of *Bacillus* spp.

**Figure 3:**
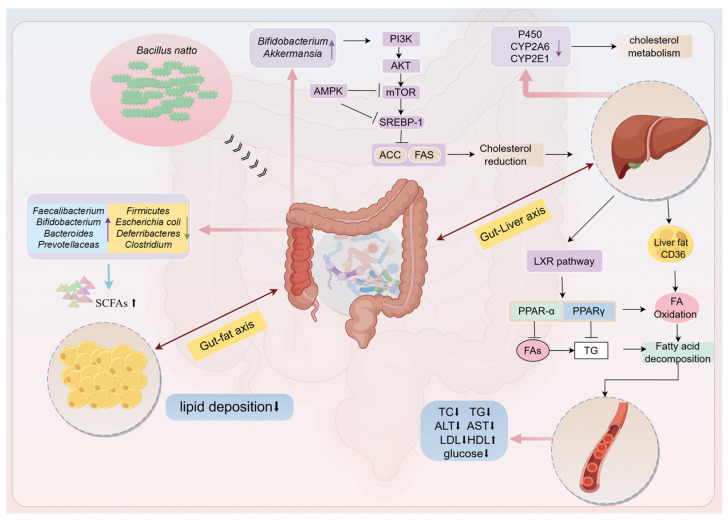
Potential mechanisms of *Bacillus subtilis* and *Bacillus natto* and their effects on intestinal metabolism. On the right side of the strain name, the SCFA, lipid deposition, P450, and TC frames, the arrows indicate an increase or decrease in content or level; other arrows merely represent the meaning of indication or connection. The arrow formed by the connection of a horizontal line and a vertical line indicates the expression of inhibition.

Collectively, BS and BN improved intestinal and systemic lipid metabolism in livestock and poultry through (Figure 3): (i) ACC-mediated suppression of hepatic fatty acid synthesis; (ii) gut microbiota remodeling favoring beneficial taxa; (iii) enhanced cholesterol and triglyceride catabolism; and (iv) induction of CYP450 enzymes promoting xenobiotic detoxification. These pleiotropic effects—particularly reduced serum lipids and fecal skatole, alongside improved meat quality—supported BS and BN as effective feed additives for sustainable production.

### 4.2. Intestinal Metabolism Regulation of BS and BN in Model Animal

Probiotics possess the capability to regulate the structure of intestinal flora and their metabolic profile, which in turn, regulate the metabolic level of the host. The consumption of BN-fermented peanut meal extracts effectively heightened leptin and lipid levels and protected the intestinal mucosa in hyperlipidaemic mice [94]. It was ascertained that BN repairs the insulin resistance by activating the PI3K/AKT signal pathway, thereby improving HFD-induced obesity in SD rats, suggested BN is a potential probiotic and anti-obesity bacterium [95]. A reduced Bacteroidetes/Firmicutes (B/F) ratio was regarded as one of the structural characteristics of intestinal flora related to chronic diseases such as hyperlipemia and obesity. The B/F ratio correlated positively with plasma glucose, triglyceride, and hepatic fatty acid synthase (FAS) levels, and negatively correlated with the relative abundance of *Lactobacillus* [96]. Feeding obese mice with γ-PGA-enriched natto led to a significant reversion of the B/F ratio and a considerable increase in the fecal bile acid and lipid levels [97]. Feeding obese mice with BN-fermented *Ruditapes philippinarum* bioactive peptides (RBPs) increased the abundance of intestinal flora associated with SCFA synthesis (such as *Bacteroidetes*, *Prevotellaceas* and *Muribaculaceae*) and regulated the abundance of intestinal flora related to intestinal inflammation (decreased the abundance of *Deferribacteres* and increased the abundance of *Alistipes*), which have weight-loss and lipid-lowering functions [98]. It has also been confirmed that the body mass and visceral fat weights were significantly decreased in obese mice fed with BN, and the effect was superior to that of *Lactobacillus plantarum*. Meanwhile, BN inhibited fatty acid synthesis and promoted hepatic fatty acid catabolism by regulating the gene expressions of peroxisome proliferators-activated receptor alpha (PPAR-α) and sterol regulatory-binding protein (SREBP-1c) [99].

The above studies fully confirm the great potential of BN as a new type of probiotic in promoting lipid metabolism and preventing obesity and other related metabolic diseases. In summary, the intestinal regulation pathways of BN in model animals which include the following: (i) increasing the abundance of beneficial bacteria such as *Bifidobacterium*, *Lactobacillus*, and *Akkermansia* activates signaling pathways such as PI3K/AKT and AMPK/SREBP-1 to increase blood lipids Metabolite levels (such as acetylcarnitine, free carnitine), reduce cholesterol levels, and alleviate host hyperlipidemia [100]; (ii) regulating the expression levels of liver PPARα, PPARγ, CD36 and FAS activates LXR-related signaling pathways, enhances fatty acid decomposition, inhibits fatty acid synthesis, reduces insulin resistance, and alleviates liver damage [95].

### 4.3. The Health Promotion Role of BS and BN in Human Intestinal Metabolism

Gut microbes are one of the most important target organs for diet regulation of human health. SCFAs are the most intensively studied metabolites of gut microorganisms. In a 4-week intervention involving 18 healthy adults, dietary supplementation with *Bacillus subtilis* DSM32315 significantly improved fasting blood glucose, circulating lipid profiles, and the satiety hormones glucagon-like peptide-1 (GLP-1), peptide YY (PYY), and fecal butyrate levels, suggesting that BS might regulate human lipid metabolism by enhancing intestinal butyrate levels. Another separate human study indicated that the serum TC and LDL levels in subjects were significantly reduced after a continuous natto diet for 2 weeks, and the mechanism needs to be further explored [101]. BS also plays a role in metabolic regulation in the treatment of intestinal diseases. After 14 days of bismuth quadruple therapy (3 times/day) for helicobacter pylori patients, probiotics composed of *Bacillus subtilis* (5.0 × 10^7^ cfu/mL) and *Enterococcus faecium* (4.5 × 10^8^ cfu/mL) were provided to the patients for 4 weeks. The results showed that probiotics did not affect the eradication rate of *Helicobacter pylori* but significantly reversed the accumulation of harmful bacteria caused by treatment such as *Shigella*. At the same time, the pathway of cofactor and vitamin metabolism in intestinal flora was enriched [102].

Despite limited human data, BS and BN improved glycemic and lipid profiles, elevated butyrate and satiety hormones, and restored antibiotic-disrupted microbiota metabolism—supporting their role in gut–liver and gut–fat axes. However, underlying mechanisms, particularly natto-mediated cholesterol reduction, and limiting factors (strain specificity, dosage, interindividual variability) warrant systematic investigation in well-controlled clinical trials.

## 5. Limitations and Future Outlook

**Figure 4:**
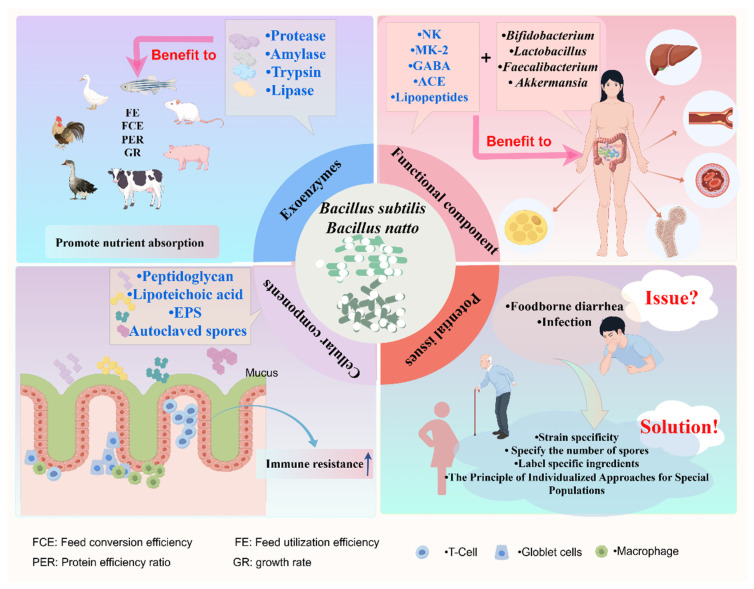
The component basis of the probiotic activity of *Bacillus subtilis* and *Bacillus natto* and the problems and challenges they face as a potential probiotic for human consumption. The upward arrow indicates causing or enhancing.

Longitudinal studies across species confirmed that BS and BN exert beneficial effects on host nutrient assimilation, lipid homeostasis, and immune modulation, primarily through gut microbiota regulation [103]. In particular, epidemiological evidence suggests that long-term natto consumption is associated with improved cardiovascular health, enhanced bone density, and better glycemic control, contributing to its reputation as a potential longevity-promoting food (Figure 4).

Despite global probiotic recognition, adverse events—particularly bacteremia in immunocompromised patients—were documented for BN [104,105,106,107]; five cases since 2000 were successfully treated with penicillin [104,105,106,107,108,109,110,111,112,113] (Table 2). These findings underscore the necessity for comprehensive studies to evaluate the safety profile, stability, and long-term therapeutic efficacy of BN. In addition, it is crucial to recognize that probiotic effects are strain-specific, meaning that different strains within the same species may exhibit distinct biological activities and health benefits [114,115,116,117]. *Bacillus natto* JLCC513 combined with ginseng soluble dietary fiber (BG) synergistically alleviated DSS-induced colitis in mice via the LPS/TLR4/NF-κB pathway and gut microbiota remodeling, supporting its potential for UC management [115]. A randomized, double-blind, placebo-controlled clinical trial (Randomized Controlled Trial, RCT) demonstrated that *B. subtilis* BS50 alleviated functional bloating through enhanced digestion and anti-inflammatory activity [116]. Conversely, a nine-strain formula (including *Bifidobacterium* and *Lactobacillus*) outperformed single-Bacillus strain probiotics and fecal microbiota transplantation (FMT) in Irritable Bowel Syndrome-Diarrhea (IBS-D) by restoring microbiota composition and anti-inflammatory metabolite networks [117]. The above results indicated that *Bacillus* species exhibit strain-specific characteristics under different intestinal pathological conditions. That is to say, not every strain of *Bacillus* can show significant effects in clinical treatment, and not every type of Bacillus is suitable to be a regulator of intestinal diseases. Notably, complex microbial interactions, including competition, inhibition, synergy, and cross-feeding phenomena, have been observed when BS is co-cultured with *Lactobacillus*, yeast, and other probiotic strains [108]. In the future, large-scale clinical studies are needed to verify the universality of multi-strain compound formulations and to further clarify the specific mechanisms of action of different strains in different intestinal environments. Systematic investigation of these interactions is essential for developing advanced probiotic formulations with enhanced fermentation capabilities and therapeutic potential.

## 6. Conclusions

*Bacillus subtilis* (BS) exhibited superior nutrient efficiency, stress resistance, and enzymatic diversity, positioning it as a leading industrial probiotic. BS spores survived gastric transit at a rate of nearly 100%, germinated in the intestine, and consumed oxygen to create an anaerobic niche favorable for *Lactobacillus* and *Bifidobacterium*—a distinct ‘oxygen bio-capture’ strategy against dysbiosis of the intestinal flora. Some of the BS strains that have undergone clear genetic testing and functional verification also held non-path Qualified Safety Presumption (QSP) by the European Food Safety Authority and have attained GRAS (Generally Recognized as Safe) status by the FDA, confirming its safety [114].

In conclusion, this review systematically established the gut-centric triple mechanism of BS and BN in nutrition, immunity, and metabolism, providing a theoretical foundation for spore probiotic development. In the future, ‘personalized’ customized *Bacillus* probiotics with clear genetic sources, safe active ingredients and strong health effects will be more in line with market demand, which is also a direction that researchers need to focus on and strive toward.

## Figures and Tables

**Table 1:** The role and mechanism of *Bacillus subtilis* and *Bacillus natto* in promoting intestinal absorption in animals and humans.

Study Object	Action Effect	Action Mechanism	Reference
Ross308 broiler chicks	Enhance the morphology of the intestine, promote the production of digestive enzymes	Enhance the expression of SGLT-1, GLUT-2, and PEPT-1 in jejunum.	[13,14]
Broiler chickens	Enhance the height and surface area of jejunal villi and augment enzyme activity	Increased in jejunal MUC2 mRNA expression, which is associated with morphological changes in small intestine	[15]
Red sea bream	Enhance feed conversion efficiency and protein efficiency ratio, promoting body weight	Improve intestinal digestive enzyme activity and the beneficial bacteria abundance (such as *Lactobacillus*).	[17,18]
*Epinephelus coioides*	Enhance the feed utilization efficiency and body weight	BS has excellent nutrient supplements and protease, lipase, amylase activity.	[20,21]
White-leg shrimp	Enhance protein utilization and intestinal immunity to promote weight gain	BS enhances the intestinal structure of shrimp and the activities of trypsin, amylase, and lipase. The improvement in growth performance stimulates the expression of immune-related genes such as serine protease, peroxinectin, and prophenoloxidase.	[22,23]
White king pigeons	Enhance the proportion of beneficial bacteria	Firmicutes phylum plays a crucial role in regulating energy and nutrient absorption	[24,25]
White-feather broilers	Enhance feed utilization efficiency of jejunal mucosa	feed conversion ratio was significantly correlated with jejunum mucosal barrier protein expression (ZO-1, claudin-1 and occludin).	[26,27]
Bamei piglets	Optimize the composition of the intestinal flora and enhance feed intake	Enhance the abundance of *Prevotellaceae*, *Rikenellaceae*, decrease intestinal permeability and increase serum AKP and total protein content.	[28,29]
White leghorn chickens	Improve intestinal villus morphology, cell area and mitosis, and increase intestinal amylase and lipase activities	Depress blood ammonia concentration, activate intestinal function.	[10,13]
Muscovy ducks	Enhance protein absorption, modulate hormone secretion, inhibit harmful bacterial (*E. coli* and *Salmonella*), and improve the duodenal structure and immune function in Muscovy ducks.	Enhance the abundance and activity of *Lactobacillus* by produce catalase and subtilisin.	[31,32]
Finishing pigs	Increase the height of ileal villi and the level of SCFAs in colon, prevent pathogen translocation	Enhance the mRNA expression levels of *claudin-1*, *mucin-1*, and *occludin* to preserve and reinforce intestinal barrier integrity, and optimize gut function.	[34]
Nursery pigs	Elevate the concentrations of fecal SCFAs and bile acids, thereby optimizing the composition and functional of intestinal flora	Upregulate the mRNA expression level of glucose transporter 2 (GLUT2) and enhances intestinal glucose absorption.	[35,36]
Holstein calves	Enhance feed conversion efficiency and promote body weight gain	BS promotes the development of rumen flora and enhances the utilization of intestinal nutrients as a probiotic.	[33,37]
Duhan hybrid lambs	Enhance growth rate and feed efficiency	BS enhanced the abundance of *Bacteroidetes* and butyrate level, which promoted polysaccharide decomposition and fiber digestion.	[38,39]
BALB/c mice	Increase the concentration of soluble Ca in small intestine	Inhibit the formation of insoluble Ca complex and increase the content of soluble Ca in intestinal lumen.	[42]
KM mice	Optimize the composition of intestinal flora and enhance the bile acid metabolism	Promote the release of 5-HT from enterochromaffin cells via the bile acid metabolism and TGR5 receptor pathway, thereby enhancing intestinal peristalsis	[10]
C57BL/6 mice	Promote body weight gain	Tryptophan produced by BS affects 5-HT signaling in the intestinal epithelium and increases intestinal peristalsis.	[43]
KM mice	Improve feed utilization, body weight, and intestinal mucosal morphology	Promote the proliferation of beneficial bacteria *Lactobacillus* and *Bifidobacterium*, and reduce the abundance of harmful bacteria *E. coli* and *Staphylococcus aureus*.	[45]
SD rat	Increase pH in the distal small intestine, enhancing calcium solubility.	Enhance small intestinal Ca absorption by prolonging intestinal transit time and increasing passive Ca transport.	[48]
Adult	Promote the digestion and absorption of protein and dietary fiber	Enhance the abundance of *Bifidobacteriaceae* and *Bacteroideaceae*, promote the generation of SCFAs.	[49,50]
Adult woman	Enhance the Ca absorption efficiency in small intestine	Activation of the MCM3-Keap1-Nrf2 signaling pathway in osteoblasts significantly enhances osteoblastogenesis, thereby promoting bone formation and maintaining skeletal health.	[50,52]

**Table 2:** Cases of human infection with *Bacillus subtilis* or *Bacillus natto* from 2000 to 2025.

Case Ref.	Age	Sex	Underlying Diseases	Chief Complaint	Diagnosis	Treatment Methods	Prognosis
[109]	41	Woman	medical history of congenital liver fibrosis	fever	Portal vein pyogenic thrombophlebitis	Meropenem, antibiotic and cefaclor	Cured
[110]	70	Man	insomnia	high fever	Pneumonia and bacteremia	Vancomycin followed by levofloxacin and systemic corticosteroid	Cured
[104]	53	Woman	a free previous medical history	fever and chills	Persistent bacteremia with liver and splenic abscesses	Vancomycin, ampicillin–sulbactam and oral amoxicillin–clavulanic acid	Cured
[106]	70	Woman	hemodialysis	fever, dyspnea, and general malaise	Multiple myeloma	Antiviral and immunotherapy for COVID-19 and Vancomycin	Cured
[111]	56	Woman	hypertension	abdominal pain	*Bacillus subtilis* variant natto Bacteremia of Gastrointestinal Origin	Ampicillin/sulbactam and antimicrobial	Cured
[107]	67	Woman	systemic scleroderma, polymyositis and reflux esophagitis	fever, headache and disturbed consciousness	Bacteremia with bacterial meningitis	Meropenem and vancomycin	Cured
[105]	65	Man	diabetes mellitus	fever and perianal pain	Sigmoid colon perforation	Abdominal drainage and antibiotics	Cured
[112]	-	Newborn infant	late premature neonate	infrequent desaturations, minimal abdominal distention and positive occult blood tests	Neonatal Sepsis	Meropenem and vancomycin, and under the new treatment	Cured
[113]	51	Man	a free previous medical history	started a month ago with recurrent episodes of left upper limb weakness	Spontaneous cerebral abscess	Surgery, antibiotic treatment and amoxicillin/clavulanic acid	Cured

Note: The references were listed in reverse-chronological order.

## Data Availability

All data generated or analyzed during this study are included in this published article.
